# A Homogalacturonan from Peel of Winter Jujube (*Zizyphus jujuba* Mill. cv. *Dongzao*): Characterization and Protective Effects against CCl_4_-Induced Liver Injury

**DOI:** 10.3390/foods11244087

**Published:** 2022-12-17

**Authors:** Shuguang Sun, Wenzhong Lan, Li Ji, Lianzhong Ai, Yan Wu, Hui Zhang

**Affiliations:** 1 Shandong Food Ferment Industry Research & Design Institute, Qilu University of Technology (Shandong Academy of Sciences), Jinan 250013, China; 2 Shanghai Engineering Research Center of Food Microbiology, School of Health Science and Engineering, University of Shanghai for Science and Technology, Shanghai 200093, China; 3 Department of Food Science & Technology, School of Agriculture and Biology, Shanghai Jiao Tong University, Shanghai 200240, China

**Keywords:** winter jujube, homogalacturonan, characterization, conformation, hepatoprotective effects

## Abstract

A homogalacturonan pectin (HG, designated as WJP-F80) was extracted from the peel of winter jujube (*Zizyphus jujuba* Mill. Cv. *Dongzao*) and separated via ethanol-graded precipitation. The structural and conformational features were elucidated through HPAEC-PAD, GC-MS, 2D NMR, and HPSEC-MALLS studies. In vivo assessments were carried out to evaluate the hepatoprotective effects of WJP-F80 against CCl_4_-induced injury of mice. Results showed that WJP-F80 was a linear 1,4-α-galacturonan with partially methyl-esterified at *O*-6 of Gal*p*A and occasionally acetylation. The Mw of WJP-F80 was determined as 45.3 kDa, the polydispersity was calculated as 1.56, and the R*g* was measured as 22.7 nm in 0.1 M NaNO_3_. The conformational analysis revealed that WJP-F80 exhibited as rigid stiff chain in low Mw range, while aggregation by self-assembly of HG chains lead to high Mw and random coil conformation. In vivo studies indicated that WJP-F80 can protect the livers of mice from acute injury induced via CCl_4_ by decreasing the serum biochemical markers of alanine aminotransferase (ALT) and aspartate aminotransferase (AST) to normal levels. This work provides a theoretical basis for the value-added deep processing of winter jujube.

## 1. Introduction

Jujube is a popular fruit of *Zizyphus jujuba* (*Z. jujuba*) Mill. (*Rhamniaceae* family) used as both medicine and food, and mainly distributes in the northwest region and Yellow River basin of China. It is a very famous fruit and herbal medicine recorded in ancient Chinese books *Huangdi Neijing* (475–221 BCE) and *Shennong Bencao Jing* (300 BCE-200 CE) [1]. It was described to possess the capacity to nourish Qi and blood, and improve the digestive system and sleep quality [2,3]. Pharmacological studies have found that jujube could exhibit protection effects on liver, immunomodulatory, sedation, anti-cancer, antioxidant, and anti-inflammatory activities due to its various chemical ingredients [4,5,6].

As one of the most important components among the functional ingredients, polysaccharides have been isolated from many cultivars of jujubes with distinct structures [1,7,8]. It was summarized that 46 polysaccharides have been characterized from jujubes by 2022. Most of these polysaccharides were extracted by hot water and further isolated by ionic exchange chromatograph (such as DEAE resin). Structural analysis indicated that polysaccharides from jujubes were mostly acidic pectin-like heteroglycan, including homogalacturonan (HG), rhamnogalacturonan-I (RG-I), and substituted galacturonans (RG-II), with molecular weight ranging from 10^4^ to 10^6^ Da [1,6]. These polysaccharide fractions always mix together to form the cell wall structure of jujubes. For example, Zhao et al. [9] isolated two acidic polysaccharides from *Z. jujuba* Mill. cv. *jinsixiaozao* Hort (Ju-B-2 and Ju-B-3) and found that Ju-B-2 was a polygalacturonan interspersed with RG-1, whereas Ju-B-3 was HG consisting of galacturonic acid. Ji et al. [10] also purified an acidic polysaccharide from *Z. Jujuba cv. Muzao* (PZMP2-2) which consisted of rhamnose, arabinose, xylose, galactose, and galacturonic acid with molecular weight of 62.73 kDa.

Polysaccharides were proved to be the main bioactive components from jujubes and exhibit many functions, such as antioxidant [11], anti-fatigue [12], immuno-regulatory [13,14], anti-inflammation [8,15], and hepatoprotective activities [16,17]. These bioactivities were considered to be related with the molecular weight, monosaccharide composition, and chemical structures of polysaccharides [1]. However, most of these studies gave focused on the cultivars of *Huizao*, *Jinsixiaozao*, *Muzao*, *Junzao*, *Ruoqiangzao*, etc., few works have investigated the structural characterization and bioactivities of glycans from the cultivar of *Dongzao*. As for pectin-like polysaccharide, galacturonic acid content, methyl ester, and *O*-acetyl groups were considered to be important for its bioactivities [18,19]. Most of the hepatoprotective pectins were reported to be acidic polysaccharides with backbone of 1,4-α-D-Gal*p*A [16,20]. It is interesting to investigate if the pure homogalacturonan with 1,4-α-D-Gal*p*A backbone possesses the hepatoprotective activity.

The cultivar of *Dongzao* (called as winter jujube in Chinese) was cultivated in China and is consumed popularly as fresh fruit or juice. During the juice processing, the peels of winter jujube are removed as byproducts, which results in waste and environmental pollution. In order to further explore the value of byproducts, the current study isolated a HG pectin from the peels of winter jujube firstly, and subjected them to chemical characterizations and in vivo assessment of protective effects against CCl_4_-induced liver injury in mice. This work promotes the value-added processing of byproducts of winter jujube, and in the meantime solves the pollution problems.

## 2. Materials and Methods

### 2.1. Materials

The peels of winter jujube as the byproduct of jujube juice were collected from Shandong Qilu Haohua Food Technology Co. LTD, Shangdong, China. The monosaccharide standard series including glucuronic acid (Glc*p*A) and galacturonic acid (Gal*p*A) were purchased from Beijing Spectroscopic Standard Technology Co. LTD (Beijing, China). Deuterium oxide (99.9% D) and sodium borodeuteride (98% D) were from Sigma-Aldrich (St. Louis, MO, USA). Unless otherwise specified, all other reagents and materials were analytical grade.

### 2.2. Extraction and Fractionation of Galacturonan Pectin from Peel of Winter Jujube

The peel of winter jujube was collected as a byproduct after the processing of jujube juice. The polysaccharide was extracted by acidic solution and ethanol precipitation. Briefly, 1000 g powder of dried peels was suspended in 2 L of HCl solution (pH 3.0), and extracted for 45–60 min at 90 °C. The extracted solution was treated with papain for 60 min at 55 °C, and decolorized by activated carbon for 30 min at 85 °C. The obtained extracts were then concentrated and precipitated with absolute ethanol to obtain crude sample designated as cWJP. The cWJP was further successively fractionated by ethanol with final concentration of 50% (F50), 65% (F65), 80% (F80), and 90% (F90) (*v*/*v*). The highest yield fraction of F80 (with yield of ~83%) was selected and deproteinized by Sevage method to obtain a homogeneous fraction of WJP-F80.

### 2.3. Monosaccharide Composition Analysis

High-performance anion-exchange chromatography (HPAEC, Dionex ICS-5000, USA) was applied to determine the monosaccharide composition of WJP-F80 according to previous the method [21]. A CarboPac™ PA20 analytical column (4 mm × 250 mm) was used for separation, and a pulsed amperometric detector (PAD) was used for detection. Polysaccharide samples (0.5 g) were firstly treated with H_2_SO_4_ (12 M, 0.5 mL) for 30 min at 25 °C. Pure water was then added to dilute H_2_SO_4_ to 2 M. The diluted solution was heated to 100 °C and hydrolyzed for 2 h. After cooling, the final hydrolyzed solution was diluted with pure water, filtrated, and injected for analysis directly. Monosaccharide standards were used to determine the type and amounts of composition. All samples were conducted in triplicate and averaged.

### 2.4. Methylation and GC-MS Analysis

Due to the high content of uronic acid, reduction was firstly conducted following the method as described before [22,23] with slight modifications. Briefly, 5 mg of sample in 2 mL of D_2_O was mixed with 1-cyclohexyl-3-(2-morpholino-ethyl) carbodiimide metho-p-toluenesulfonate (CMC, 50 mg) thoroughly. During the mixing process, the pH of the solution was maintained at ~4.75 using 0.1 M HCl in D_2_O. After stirring for 1 h, NaBD_4_ (800 mg) in D_2_O was added drop by drop in 30 min, and HCl in D_2_O was used to adjust pH~4.0. The reduced sample (R-WJP-F80) was then dialyzed against water, freeze-dried, and dehydrated in a vacuum oven. FT-IR was applied to assess the reduction degree to ensure that the –COOH group (around at 1700 cm^−1^) disappeared completely.

Methylation process of R-WJP-F80 followed a previous method by Cui [23] with slight modification [21]. The reduced sample was dried thoroughly, then dissolved in anhydrous 1 mL of DMSO to obtain a clear solution. Dried NaOH (powder, 20 mg) was added to the solution to provide alkaline environment. For the methylation reaction, 0.3 mL of methyl iodide was added to the solution drop by drop for 30 min in an ice-water bath, then reacted at room temperature for 2.5 h with constant stirring. CH_2_Cl_2_ (1 mL) was added to the reaction solution to extract the methylated sample, and the extracted solution was washed with water three times. The final CH_2_Cl_2_ extract was injected to a Na_2_SO_4_ column (0.5 × 15 cm) to remove water, and dried by a blow of nitrogen. The dried sample was treated with 4 M trifluoroacetic acid (TFA) for hydrolysis. The partial methylated alditol acetates (PMAA) were obtained followed by reduction via NaBD_4_, and acetylation with acetic anhydride. GC-MS system (THERMO 1310 GC-ISQ LT MS, USA) equipped with a TG-200MS capillary column (30 m × 0.25 mm, 0.25 mm film thickness, Thermo Fisher, USA) was applied for analysis according to a temperature program (from 160 to 210 °C at 2 °C/min, and then 210–240 °C at 5 °C/min).

### 2.5. NMR Study

Before NMR study, dried WJP-F80 was dissolved in D_2_O and lyophilized, which was repeated three times with the aim to exchange the deuterium. The ^1^H (600.10 MHz) and ^13^C (151.01 MHz) NMR spectra were recorded for deuterated sample on a Bruker AVIII 600 NMR spectrometer (Brucker, Rheinstetten, Germany) at 300 K. The 2D NMR were conducted to record the ^1^H/^1^H correlation (DQF-COSY) and ^1^H/^13^C correlation (HSQC). Heteronuclear multiple-bond correlation (HMBC) experiments were also conducted to record the ^1^H/^13^C remote correlation. Trimethylsilyl propionate (TSP) in D_2_O was sued as external standard to correct the chemical shifts for ^1^H (0.0 ppm).

### 2.6. Determination of Molecular Parameters

High-performance size-exclusion chromatography system (HPSEC) was applied for the molecular parameter analysis of WJP-F80 according to our previous methods [24]. Sample was dissolved in NaNO_3_ solution (0.1 M) and filtered for injection. An OHpak SB-803 HQ column and an OHpak SB-805 HQ column (8 mm × 300 mm, Shodex, Tokyo, Japan) in series were used for separation, and 0.1 M NaNO_3_ solution containing 0.02 wt% NaN_3_ was applied as eluent with a flow rate of 0.6 mL/min. Three detectors, a multi-angle laser light scattering detector (DAWN HELEOS-II, MALLS), a differential pressure viscometer (ViscoStar III, DP), and a refractive index detector (Optilab T-Rex, RI) (Wyatt Technology, Santa Barbara, CA, USA) in series were equipped with the HPLC system. For the data collection and analysis, ASTRA 7.1.3 software (Wyatt Technology, Santa Barbara, CA, USA) was applied, and a DN/DC value of polysaccharide sample was adopted as 0.145 for the calculation of molecular parameters [25].

### 2.7. Animal Grouping and Experimental Design

A total of 60 male Kunming mice (weight 16~20 g) were purchased from Pengyue Experimental Animal Breeding Co. Ltd. (Jinan, China). They were housed in a pathogen-free room with temperature of 20~25 ºC and humidity of 40~70%. After acclimation for 4 days, they were divided into five groups averagely: (I) normal control group, mice provided pure water instead of WJP-F80 and CCl_4_; (II) CCl_4_ model control group, mice provided pure water instead of WJP-F80 and then treated with CCl_4_; and (III-V) WJP-F80-treated groups, mice administered WJP-F80 at different doses (100, 200, and 400 mg/kg·BW for group III, IV, and V, respectively), and then treated with CCl_4_. The polysaccharide sample was dissolved in pure water to final concentrations of 10, 20, and 40 mg/mL for group III, IV, and V, respectively. All animal treatments were administered with certain volume of sample solution (approximately 200 μL) by intragastric gavage (stainless steel needle size: HL-GW-9 with inner diameter of 0.5 mm) for 28 consecutive days. After the last treatment, CCl_4_ solution (12.5 mg/kg in soybean oil) was intraperitoneal injected into mice in groups II-V at a dose of 0.01 mL/g·BW to induce acute liver injury, while group I was injected with saline solution. Finally, all the animals were sacrificed and the corresponding blood and livers were obtained immediately for further investigation. The current project involving animals followed the Chinese legislation on the use of laboratory animals, which were approved by the Ethics Committee of Qingdao Sci-Tech Innovation Co., Ltd., Qingdao, China (No. IACUC-2022-0202).

### 2.8. Biochemical Examinations and Histopathological Images

All mice were weighed at 24 h after each administration. The collected blood samples were centrifuged immediately and serum was obtained. The levels of ALT and AST in serum, which was considered as the biochemical markers of hepatic damage, were determined according to the method of Reitman–Frankel [26]. All the measurements were repeated for three times, and the results were averaged from each individual sample.

The collected liver tissues were preserved in paraformaldehyde solution (4% *v*/*v*, pH 7.4) at 4 °C for 24 h, then dehydrated by ethanol and cleaned by xylene. The treated liver tissues were embedded in paraffin wax and sliced by microtome. The obtained slices were examined using a light microscope after staining by hematoxilen and eosin (H&E stain).

### 2.9. Statistical Analysis

All the measurements in the current study were conducted in triplicate, the results were shown in means ± standard deviation (SD). An analysis of variance (ANOVA) was applied for significance analysis within *p* < 0.05.

## 3. Results and Discussions

### 3.1. Fractionation and Monosaccharide Composition

The yield rate of cWJP was 3.2% by dry weight. After ethanol precipitation, the recovery of WJP-F80 fractionated from cWJP was up to 83% (*w*/*w*). HPAEC-PAD results indicated that WJP-F80 was a homogalacturonan (HG) consisting of galacturonic acid, with total sugar content of 98% (*w*/*w*) (Figure S1 and Table S1). It has been reported that polysaccharides from jujube were pectin-like glycan mainly composed of galacturonic acid, and different neutral sugar of arabinose, galactose, glucose, mannose, and rhamnose, etc. [6]. Ji, et al. [27] isolated a galacturonic acid-rich polysaccharide from *Muzao* which was identified to be composed of rhamnose, arabinose, galactose, and galacturonic acid at a molar ratio of 1.74: 2.00: 1.00: 18.69. Two polysaccharides isolated from *Jinsixiaozao* were reported to be a polygalacturonan with 7.49% methoxylation and a polygalacturonan interspersed with rhamnogalacturonan in the backbone, respectively [9]. An 1,4-galacturonan was also identified from *Junzao* which possessed excellent anti-inflammatory effects in vitro [15]. However, no study has been reported about the polysaccharides from the peel of *Dongzao*, and the current isolated HG fraction needed to be further elucidated its structure and bioactivities in order to better use of the peel byproducts.

### 3.2. Structural Characterizations

The structural information including linkage patterns and sequence of sugar residues in WJP-F80 were analyzed through methylation and NMR studies. Due to the high content of uronic acid, chemical reduction of carboxyl groups was firstly conducted, then methylation was applied to WJP-F80 and GC-MS was used to analyze the linkage patterns and corresponding contents of sugar residues. The reduced WJP-F80 showed that the main PMAAs were of 2,3,6-Me_3_-Gal*p* and 2,3,4,6-Me_4_-Gal*p*, which indicated that WJP-F80 was mainly composed of 1,4-linked Gal*p*A (89.06%) and T-Gal*p*A (10.94%, including the reducing and non-reducing terminal residues) (Table S2). No branch residue was detected from the GC-MS results. These data suggested that WJP-F80 was a linear pectin-like galacturonan without branches. The relative high content of terminals might be due to the degradation of polysaccharide during methylation process which shortened the chain length of WJP-F80.

**Figure 1:**
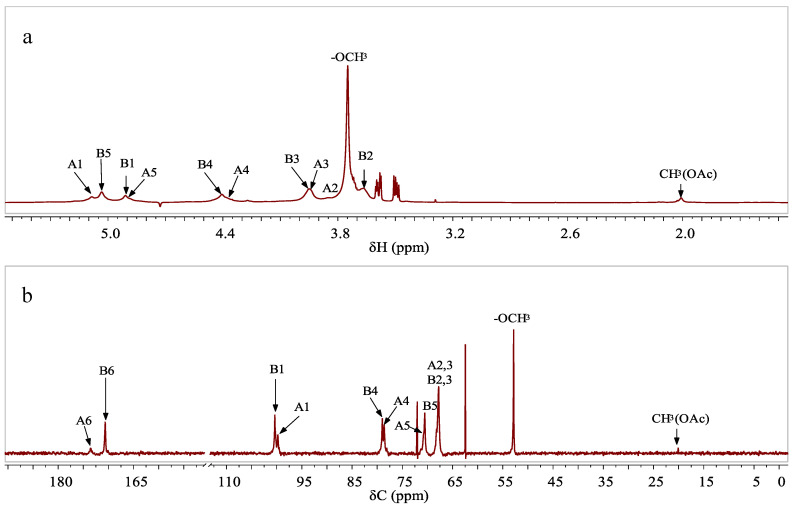
The ^1^H (**a**) and ^13^C (**b**) NMR spectrum of WJP-F80 (D_2_O, 300 K).

The 1D & 2D NMR were further used to verify the sugar units of WJP-F80. According to ^1^H NMR of WJP-F80 in D_2_O (Figure 1a), the anomeric proton signals at *δ* 5.08 and 4.91 ppm corresponded to the residues of →4)-α-Gal*p*A-(1→ (assigned as residue A) and →4)-α-Gal*p*A6Me-(1→ (assigned as residue B), respectively [28]. The signal at *δ* 3.75 ppm was assigned to the proton of methoxyl group (–OCH_3_), and the signals at *δ* 2.01 ppm was suggested to the proton of methyl of *O*-acetyl groups (CH_3_(OAc)). From the ^13^C NMR (Figure 1b), the signals at *δ* 20.08 and 52.85 ppm confirmed the presence of –OAc and –OCH_3_ groups in WJP-F80 [29]. Signals at *δ* 173.46 and 170.66 ppm were assigned to the carbon signals of carboxyl group in α-D-Gal*p*A (A6), and methyl-esterified carboxyl group in α-D-Gal*p*A(-OMe) (B6), respectively [9,30].

**Figure 2:**
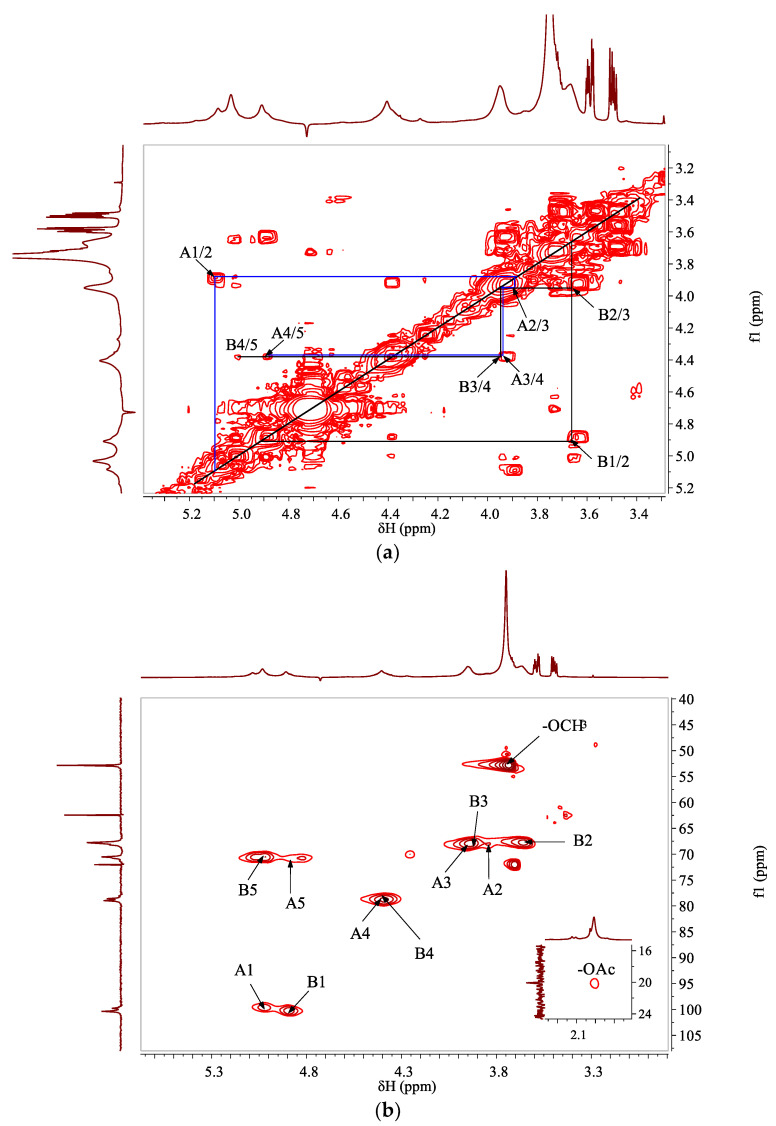
**The**^1^H/^1^H DQF-COSY (**a**) and ^1^H/^13^C HSQC (**b**) correlation spectra of WJP-F80 (D_2_O, 300 K).

The chemical shifts of non-anomeric protons of →4)-α-Gal*p*A-(1→ (residue A) and →4)-α-Gal*p*A6Me-(1→ (residue B)) were fully assigned according to DQF-COSY (Figure 2a). The corresponding carbon/proton correlations were determined via HSQC spectra (Figure 2b). Two anomeric carbon/proton correlation signals at *δ* 99.77/5.08 and 100.37/4.91 ppm were assigned to residues of A and B, respectively. The non-anomeric carbons of different residues were further verified from HSQC spectra based on the proton information of DQF-COSY and previous literatures [9,30,31], and the results were listed in Table 1. It should be noted that the proton chemical shifts at around *δ* 3.58 and 3.50 ppm, and carbon signals at *δ* 62.45 and 72.02 ppm were detected in ^1^H and ^13^C spectra; however, no correlations were found from the HSQC spectrum. These intense signals were ascribed to non-identified small molecules which might conjugate with polysaccharide and need to be further investigated.

**Figure 3:**
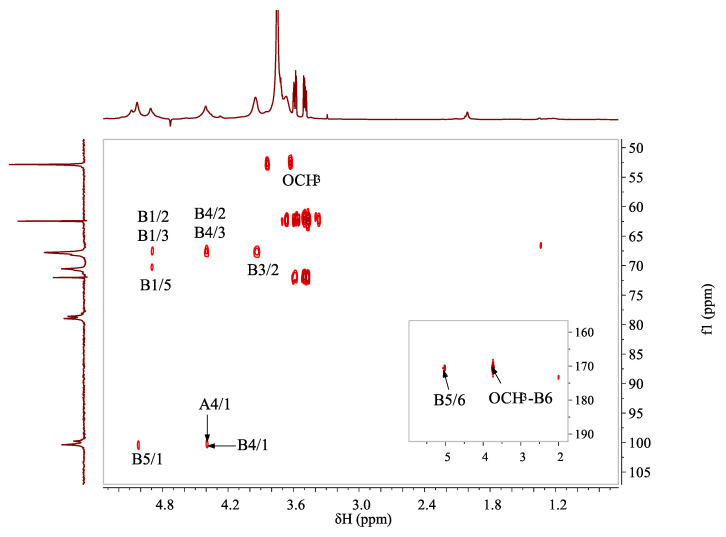
The ^1^H/^13^C HMBC correlation spectrum of WJP-F80 (D_2_O, 300 K).

HMBC spectrum (Figure 3) was applied to deduce the sequences of different sugar residues. Due to the high degree of methylation, the signals of residue B were more pronounced in the spectra than residue A. Connectivity between C1 and H4 of residue B (*δ* 100.37/4.40 ppm), as well as C1 and H4 of residue A (*δ* 99.77/4.38 ppm), were found, indicating that the linear backbone of WJP-F80 was of partially methyl-esterified α-(1→4)-galacturonan. The correlations between H5 and C6 of residue B (*δ* 5.03/170.66 ppm), and H of –OMe and C6 of residue B (*δ* 3.75/170.66 ppm) indicated that the methyl groups were linked to C-6 of residue B. All the above results indicated that WJP-F80 was a linear chain of partially methyl-esterified and/or probably acetylated 1,4-α-galacturonan.

### 3.3. Molecular Parameters Analysis of WJP-F80

**Figure 4:**
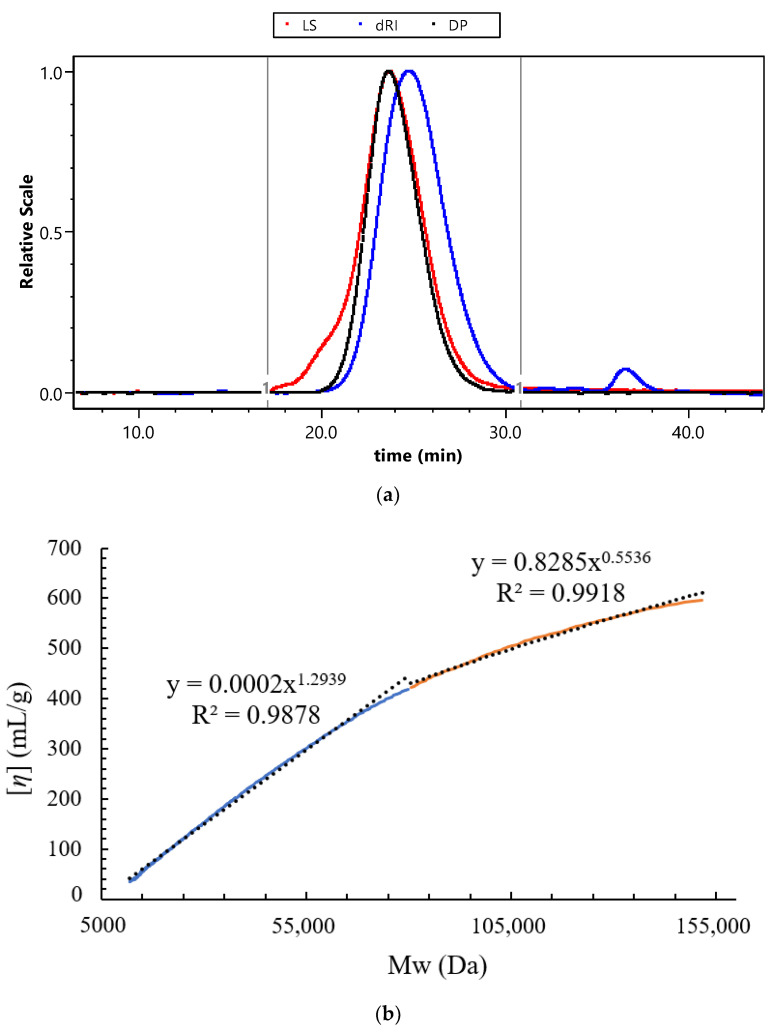
HPSEC profiles of WJP-F80 in 0.1 M NaNO_3_ solution (**a**, the signal of LS detector was from the angle of 90°) and the plots of Mw vs. [*η*] in the Mw range of 11.9–79.8 kDa and 80.5–151.7 kDa (**b**).

The HPSEC coupled with MALLS, DP, and RI detectors was conducted to analyze the molecular parameters and solution properties of WJP-F80. As shown in Figure 4a, a major single peak detected from RI profile indicated that WJP-F80 was homogeneous on molecular weight distribution. The weight-averaged molecular weight (Mw) and number-averaged molecular weight (Mn) were measured as 45.3 kDa and 29.0 kDa, respectively (Table S1). The relative low polydispersity index (Mw/Mn, 1.56) revealed that polysaccharide molecules were well-dispersed. The radius of gyration (R*g*) was calculated as 22.7 nm, and the intrinsic viscosity ([*η*]) was of 20.7 mL/g for WJP-F80, which indicated that the polysaccharide chain of WJP-F80 was extended stiff [32].

Mark–Houwink equation ([*η*] = *kMw^ɑ^*), which described the relationship between Mw with [*η*], was applied to evaluate the chain conformation of WJP-F80. The parameters of *k* and *α* in the equation are related to the conformation of polymer chains in solution. It is well-known that the exponent *α* reflects a rigid sphere in a good solvent when the value less than 0.5, a random coil when in the range of 0.5~0.8, and a rigid or rod-like conformation (stiff chain) when in the range of 0.8~2.0 [32]. As shown in Figure 4b, two linear regressions were fitted to the double logarithmic curves of WJP-F80 as decreased slope observed with increasing Mw. The slopes (*α*) were calculated as 1.29 and 0.55 in the Mw range of 11.9–79.8 kDa and 79.8–151.7 kDa, respectively. This revealed that WJP-F80 exhibited rigid stiff chain conformation in low Mw range, which was consistent with the previous report [33]. However, the results revealed the random coil conformation in high Mw range of WJP-F80, which might be due to the small amount of aggregates by self-assembly of HG chains [34,35].

### 3.4. Effects of WJP-F80 on Serum ALT and AST Levels in Mice

**Figure 5:**
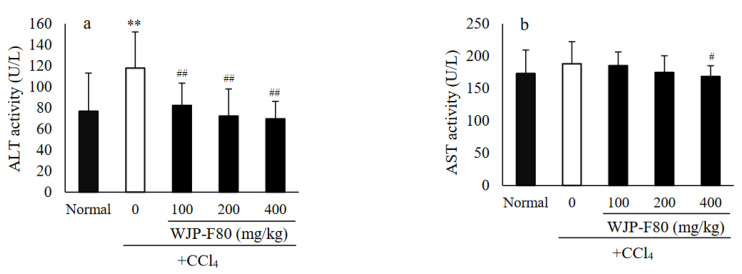
Protective effects of WJP-F80 on serum ALT (**a**) and AST (**b**) activities after administration of CCl_4_ in mice. Animals were provided either different concentration of WJP-F80 solution (Group III-V) or water (normal and CCl_4_-model groups) once daily for 28 days ahead of the simplex treatment of CCl_4_. Values are expressed as means ± SD for 12 mice in each group. Compared with normal group, ** *p* < 0.01. Compared with the CCl_4_-model group, ^#^ *p* < 0.05, ^##^ *p* < 0.01.

It has been reported that polysaccharides from jujube exhibited an important protective effect on liver [16,17]. In this study, the protective effects of WJP-F80 against the CCl_4_-induced acute hepatotoxicity were investigated in vivo. It was reported that the administrated CCl_4_ can catalyze by cytochrome P450 to form a highly reactive trichloromethyl free radical (CCl_3_), and then transfer to trichloromethyl peroxy radical (CCl_3_OO) which was considered as the precursor of lipid peroxidation [36]. The CCl_3_OO-induced lipid peroxidation resulted in the injury of liver, and consequently promoted the release of ALT and AST [37]. Therefore, the serum biochemical markers of ALT and AST are both important indicators of liver injury. As displayed in Figure 5, enzymatic activities of ALT (Figure 5a) and AST (Figure 5b) in the normal mice were determined as 76.92 ± 36.26 and 173.25 ± 56.42 U/L, respectively. After administration of hepatotoxic CCl_4_, the levels of ALT and AST elevated to 117.92 ± 34.34 and 188.08 ± 86.92 U/L in the CCl_4_-model group, thereinto, the level of ALT increased to nearly two times that of the normal group (*p* < 0.01). The prophylactic treatment with WJP-F80 before CCl_4_ administration showed the ability to decrease the levels of both ALT and AST in a dose-dependent manner by comparing with the CCl_4_-model group. At a dose of 100 mg/kg·BW, the ALT level decreased sharply to 82.42 ± 21.19 U/L (*p* < 0.01), while the AST levels had no significant statistical difference (*p* > 0.05). As the dose of WJP-F80 increased to 200 mg/kg·BW, the activities of ALT and AST were close to those of the normal control group. At the high dose group (400 mg/kg·BW), both levels of ALT and AST showed significant decrease by comparing with the CCl_4_-model group (*p* < 0.01 for ALT, and *p* < 0.05 for AST). These results implied that WJP-F80 can protect the livers of mice from acute injury induced via CCl_4_.

### 3.5. Histopathological Examination

**Figure 6:**
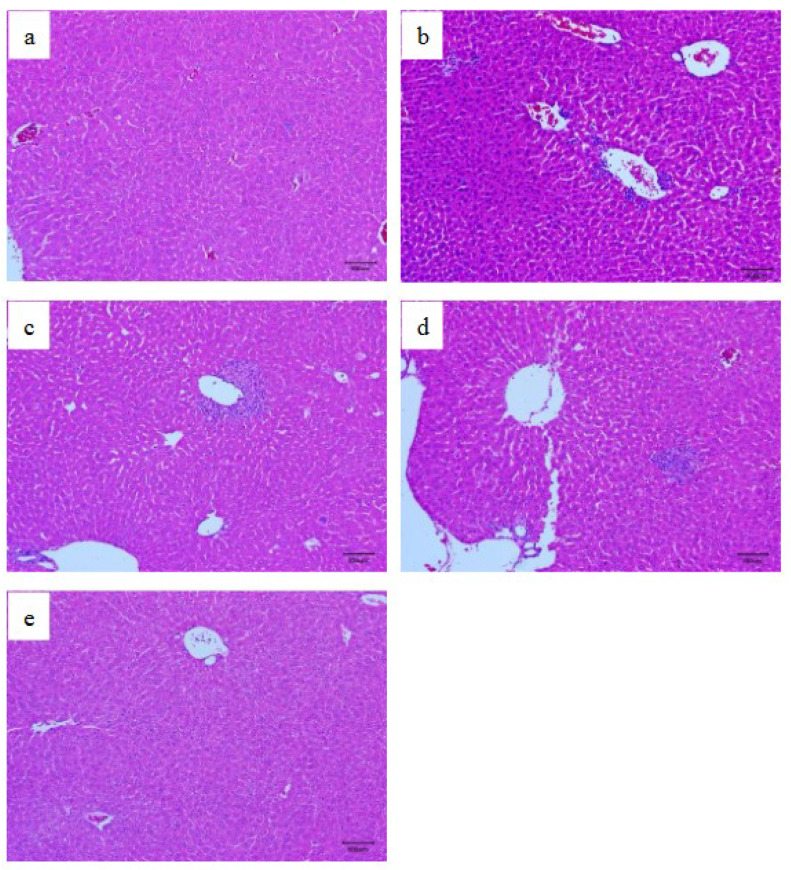
Hepatic histopathology images of normal (**a**), CCl_4_-model (**b**), and WJP-F80-pretreated (**c**–**e**) mice slices (original magnification of 100×). Liver slices were derived from normal mice (**a**), CCl_4_-model group (**b**), WJP-F80 pretreated groups at concentration of 100 (**c**), 200 (**d**) and 400 (**e**) mg/kg·BW. The mice slices were prepared by using a microtome and stained with H&E.

The hepatic histopathology was further applied to evaluate the protective effects of WJP-F80 on CCl_4_-induced liver injury. The histopathological images of liver slices selected from different groups are shown in Figure 6. The liver sections from the normal group exhibited a normal cellular architecture with healthy hepatic cells, central veins, and sinusoidal spaces (Figure 6a). However, most of the liver slices from CCl_4_-model group presented hepatocellular necrosis in extensive areas, and inflammatory cell infiltration, swelling, and vacuolation of hepatocytes were observed as shown in Figure 6b. In contrast, the pretreatment with WJP-F80 exhibited a dose-dependent improvement in the liver histopathology against CCl_4_-induced histological alteration (Figure 6c–e). Only slight inflammatory cells infiltration was found in some liver slices from WJP-F80 pretreated mice at a low concentration (Figure 6c,d). High dose treatment of WJP-F80 exhibited the ability to protect the normal liver architectures without cell necrosis and inflammatory infiltration (Figure 6e). These results were consistent with the serum biochemical marker levels and indicated that WJP-F80 can prevent the acute hepatotoxicity induced by CCl_4_ in mice.

It was reported that oxidative stress caused by free radicals was one of mechanisms for hepatotoxicity induced by CCl_4_; therefore, antioxidants can interfere with the lipid peroxidation and reduce the toxicity of CCl_4_ [38]. Uronic acid residues in polysaccharides were found to be important for their biological activities, and richer uronic acids showed better antioxidant activities [1,39]. Though only a few studies have demonstrated the direct hepatoprotective effects of the polysaccharides from *Z. jujuba* fruit, the high content of galacturonic acid in these polysaccharides implied that they can exhibit the hepatoprotective effects by inhibiting oxidative stress. In the current study, the ability of WJP-F80 to prevent liver injury induced by CCl_4_ were also considered to correlate with its HG composition which can scavenge the free radicals and inhibit the lipid oxidation process in liver organ. In addition, the high degree of methylation of WJP-F80 which provided hydrophobic interactions would also contribute to the metabolism of lipids and protect the liver from injury. However, more detailed studies are required to clarify the mechanisms and relationship between structure and hepatoprotective activities of *Z. jujuba* polysaccharides.

## 4. Conclusions

In the current study, a pectin-like HG was isolated from the peel of winter jujube (WJP-F80) and characterized to be a linear 1,4-α-galacturonan with partially methyl-esterified at *O*-6 of GalA and occasionally acetylation. The Mw of WJP-F80 was determined as 45.3 kDa, with polydispersity (Mw/Mn) of 1.56, R*g* of 22.7 nm, and [*η*] of 20.7 mL/g in 0.1 M NaNO_3_. Conformational analysis revealed that WJP-F80 exhibited rigid stiff chain conformation in low Mw range, while aggregation by self-assembly of HG chains lead to high Mw and random coil conformation. Furthermore, in vivo studies indicated that WJP-F80 exhibited the protective effect against the CCl_4_-induced acute hepatotoxicity in mice by decreasing the serum biochemical markers of ALT and AST to normal levels and preventing liver injury. As a bioactive ingredient from the peels of winter jujube, the polysaccharide extracts can be exploited as dietary ingredients for alternative daily supplements to protect live from injury. Further work will focus on the study of hepatoprotective pathway of WJP-F80 and establishing the structure–function relationship with the aim to elucidate the mechanism of hepatoprotective bioactivity of WJP-F80.

## Figures and Tables

**Table 1:** The ^1^H and ^13^C NMR chemical shifts (ppm) of WJP-F80 (D_2_O, 300 K).

Code	Sugar Residue	H1/C1	H2/C2	H3/C3	H4/C4	H5/C5	C6	-OCH_3_	-OAc
A	→4)-α-Gal*p*A-(1→	5.08/99.77	3.85/68.18	3.94/68.18	4.38/78.60	4.89/70.89	173.46		
B	→4)-α-Gal*p*A6Me-(1→	4.91/100.37	3.67/67.76	3.96/68.18	4.40/78.97	5.03/70.46	170.66	3.75/52.85	2.01/20.08

## Data Availability

Data is contained within the article or supplementary material.

## Supplementary Materials

The following supporting information can be downloaded at: https://www.mdpi.com/article/10.3390/foods11244087/s1, Figure S1: Chromatograms of HPAEC-PAD for monosaccharide standard mixture (a) and sample of WJP-F80 after acid hydrolysis (b). The standard peaks from left to right in (a) are fucose (Fuc), rhamnose (Rha), arabinose (Ara), galactose (Gal), glucose (Glc), mannose (Man), fructose (Fru), ribose (Rib), galacturonic acid (GalA), and glucuronic acid (GlcA); Table S1: The composition and molecular parameters of WJP-F80; Table S2: Linkage patterns and corresponding percentage content of sugar residues in WJP-F80 from the methylation and GC-MS analysis.
