# Supplementary material for: A Homogalacturonan from Peel of Winter Jujube (Zizyphus jujuba Mill. cv. Dongzao): Characterization and Protective Effects against CCl4-Induced Liver Injury
Source: Foods. 2022 Dec 17;11(24):4087. doi: 10.3390/foods11244087 (PMC9778428; doi:10.3390/foods11244087)
Supplement: Supplementary file 1 [file foods-11-04087-s001.zip › foods-2037557-supplementary.pdf]

Supplementary data

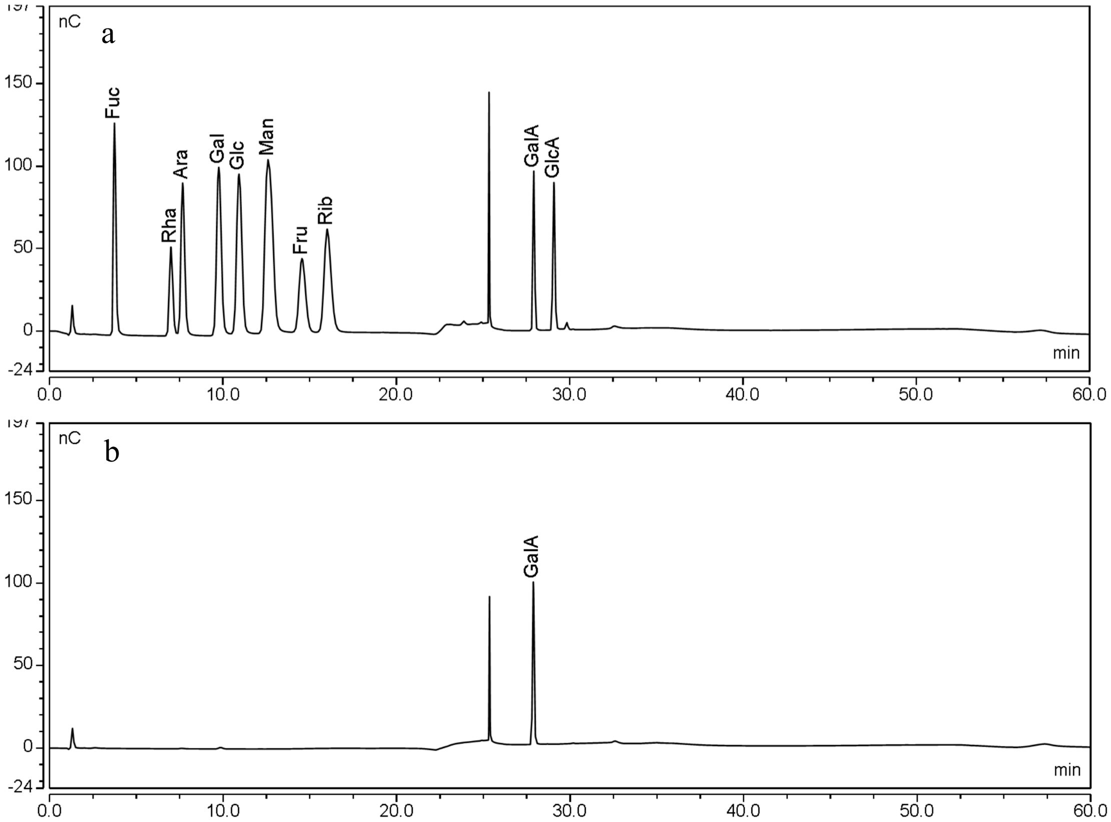

Figure S1. Chromatograms of HPAEC-PAD for monosaccharide standard mixture (a) and sample of WJP-F80 after acid hydrolysis (b). The standard peaks from left to right in (a) are fucose (Fuc), rhamnose (Rha), arabinose (Ara), galactose (Gal), glucose (Glc), mannose (Man), fructose (Fru), ribose (Rib), galacturonic acid (GalA), and glucuronic acid (GlcA).

Table S1. The composition and molecular parameters of WJP-F80

Sample	Sugar content (%)	Monosaccharide composition	Mn (kDa)	Mw (kDa)	R*g* (nm)	[*η*] (mL/g)
WJP-F80	98.0	GalA	29.0	45.3	22.7	20.7

Table S2. Linkage patterns and corresponding percentage content of sugar residues in WJP-F80 from the methylation and GC-MS analysis

RT (min) [a]	PMAAs	Linkage pattern	Percentage (%) [b]	Major m/z
12.235	2,3,4,6-Me_4-Gal*p*A	Gal*p*A-(1→	10.94	43,73,89,102,118,131,147,162,163,207
13.180	2,3,6-Me_3-Gal*p*A	→4)-Gal*p*A-(1→	89.06	43,87,99,102, 18, 29,162,175,235

[a] RT: Retention time

[b]: The percentages of each sugar residue were calculated according to the peak areas from the total ion chromatograph of PMAAs.
